# Stroke Demographics, Risk Factors, Subtypes, Syndromes, Mechanisms and Inter-Ethnic Differences between Chinese, Malays and Indians in Singapore—A Hospital-Based Study

**DOI:** 10.3390/jcdd11060180

**Published:** 2024-06-12

**Authors:** Narayanaswamy Venketasubramanian

**Affiliations:** Raffles Neuroscience Centre, Raffles Hospital, Singapore 18870, Singapore; drnvramani@gmail.com

**Keywords:** stroke, demographics, risk factors, subtypes, syndromes, mechanisms, Chinese, Malays, Indians

## Abstract

Disparities in stroke may be due to socioeconomics, demographics, risk factors (RF) and ethnicity. Asian data are scant. This retrospective hospital-based study aimed to explore demographics, RF, stroke subtypes and mechanisms among the Chinese, Malays and Indians in Singapore. Stroke was subtyped into haemorrhagic stroke (HS) and ischaemic stroke (IS). For IS, the clinical syndrome was classified using the Oxfordshire Community Stroke Project (OCSP) classification while the stroke mechanism was categorised using the Trial of Org 10172 in Acute Stroke Treatment (TOAST) classification. During the study period 1 June 2015 to 31 December 2023, data were collected on 1165 patients, with a mean age of 65.6 ± 12.9 yr; 47.4% were female, 83% were Chinese and hypertension (63.5%) and hyperlipidaemia (60.3%) were the most common RF. HS comprised 23.5% (95%CI 21.1–26.1%) (intracerebral 21.7%, subarachnoid 1.3%) of the patients, while IS comprised 76.5% (95%CI 73.9–78.9%) (small artery occlusion 29.0%, cardioembolism 13.3%, large artery atherosclerosis 9.4%, stroke of other determined aetiology 6.2%, stroke of undetermined aetiology 18.6%); 55% of patients had lacunar syndrome. A multivariable analysis showed that HS was associated with ethnicity (*p* = 0.044), diabetes mellitus (OR 0.27, 95%CI 0.18–0.41, *p* < 0.001) and smoking (OR 0.47, 95%CI 0.34–0.64, *p* < 0.001). There were no significant inter-ethnic differences by the OCSP (*p* = 0.31) or TOAST (*p* = 0.103) classification. While differences in stroke subtype in Asia may be due to RF, ethnicity has a role. More studies are needed to further explore this.

## 1. Introduction

Stroke is a major cause of death and disability globally. The Global Burden of Disease (GBD) study reported that in 2021 there were 11.9 million (95%UI 10.8–13.2) incident strokes, 93.8 million (95%UI 89–99.3) prevalent strokes, 7.25 million (95%UI 6.60–7.82) deaths due to stroke and 160 million (95%UI 148–172) Disability-Adjusted Life Years (DALYs) lost from stroke [1]. With growing and ageing of the world population, the burden is likely to increase.

The GBD study also revealed differing stroke burdens in different countries. The highest age- and sex-standardised stroke incidence was in China (226.4/100,000, 95%CI 210.8–245.0), while the highest age- and sex-standardised DALYs due to stroke was in Papua New Guinea (5091.4/100,000, 95%CI 4299.2–5991.2). The corresponding lowest rates were in New Zealand (79.0/100,000, 95%CI 73.7–84.5) and Iceland (422.2/100,000, 95%CI 384.5–462.7) [2]. These disparities have been attributed to differing socioeconomic statuses of countries, which are strongly associated with the occurrence and management of modifiable risk factors [3]; poorer acute healthcare for stroke and poorer stroke awareness may also play a part [1,4].

Globally, among the subtypes of stroke, ischaemic stroke (IS) comprises 65.5% of all incident strokes; intracerebral haemorrhage (ICH) comprises 28.9% and subarachnoid haemorrhage (SAH) comprises 5.9% [1]. The highest age- and sex-standardised incidence of ICH was in Mongolia (102.9/100,000, 95%CI 96.0–110.5), while the lowest was in Switzerland (14.1/100,000, 95%CI 12.7–15.6) [2]. An increased risk of ICH was seen in low-income to upper-middle-income countries—this may be related to the relatively high clinical significance and population-attributable risk of hypertension in these countries [1]. Geographically, the highest incidence of ICH was in Oceania, East Asia, Central Asia and Southeast Asia, while the lowest was in Western Europe and Australasia [2,5]. These ecological studies suggest that there may be inter-ethnic differences in stroke subtypes.

Asia, where 60% of the world’s population lives, has a particularly high burden of stroke [2]. A review of hospital-based stroke registries in Asia revealed that the frequency of ICH was as high as 49.1% in India and 47.2% in Vietnam but as low as 16.1% in Taiwan and 17.8% in Japan; SAH ranged from 1.3% in Vietnam to 8% in Nepal—there was a wide range of IS mechanisms and vascular risk factors [6]. These studies involve populations from different countries and provide information on stroke in that country—they allow indirect comparisons of stroke patterns. However, each investigates a relatively ethnically homogenous population with little inter-ethnic comparative data—thus they are unable shed much light on the impact of ethnicity on stroke. Singapore [7] and Malaysia [8], located in the heart of Southeast Asia, have populations comprising different ethnicities—largely Chinese, Malays and Indians. The hospital-based Singapore Stroke Registry [9] and National Neurology Registry of Malaysia [10] are able provide some insights into the demographics, risk factors and stroke subtypes of the main ethnic groups, but they have not performed detailed statistical analysis comparing the ethnicities. Also, neither has data on IS mechanisms or syndromes. There is, thus, a knowledge gap on ethnic differences in stroke, particularly in Southeast Asia.

This study was, thus, performed to study demographics, risk factors, stroke subtypes and IS syndromes and mechanisms among Chinese, Malays and Indians admitted to Raffles Hospital, Singapore, and to explore inter-ethnic differences. The results of this study provide novel data that allow direct and detailed inter-ethnic comparison of hospitalised stroke patients, which may impact on stroke prevention and management in specific ethnicities.

## 2. Materials and Methods

### 2.1. Study Setting

Singapore is an island city-state of 5.92 million people [7]. Heavily-subsidised affordable healthcare at the primary and hospital level is widely available [11], including at acute care hospitals, step-down community care facilities and some nursing homes. Private healthcare is also available, as is healthcare insurance. Raffles Hospital is a multi-specialty hospital located at the edge of the city centre, Singapore [12]. It caters to private and public patients.

### 2.2. Inclusion Criteria

Data on patients diagnosed with acute stroke were entered into a database if they were admitted between 1 June 2015 and 31 December 2023 and met the following criteria:

1. Clinical diagnosis of stroke—rapidly developing clinical signs of focal (at times global) disturbance of cerebral function lasting more than 24 h or leading to death with no apparent cause other than that of vascular origin [13]. Stroke was also diagnosed among those with focal or global neurological symptoms lasting <24 h if there was imaging evidence of brain tissue injury due to a vascular cause [14].
2. Admitted within 7 days of symptom onset.
3. Had undergone Computed Tomography (CT) or Magnetic Resonance Imaging (MRI) of the brain.

### 2.3. Data Collected

Data were collected on patient age at the time of stroke, sex, ethnicity (Chinese, Malay, Indian, others), history of vascular risk factors, results of imaging to determine stroke subtype, IS mechanism and IS syndrome.

### 2.4. Risk Factors

Hypertension (HT) was diagnosed if there was a history of hypertension or the patient was ever prescribed blood-pressure-lowering medications. Diabetes mellitus (DM) was diagnosed if there was a history of diabetes mellitus or the patient was ever prescribed glucose-lowering medications. Hyperlipidaemia (HL) was diagnosed if there was a history of hyperlipidaemia or the patient was ever prescribed lipid-lowering medications. Smoking was diagnosed if there was a history of ever having smoked. Heart disease (HD) was diagnosed if there was a history of heart disease or the patient was ever prescribed medications or underwent interventions for HD. Previous cerebrovascular disease (pCeVD) was diagnosed if there was a history of stroke or transient ischaemic attack or the patient was ever prescribed medications or underwent interventions for pCeVD. A history of intake of oral contraceptive pills (OCP) or hormone replacement therapy (HRT) was asked of the females.

### 2.5. Brain Imaging and Stroke Subtype

All patients underwent CT or MR imaging of the brain to determine the stroke subtype [14]. IS was diagnosed if there was a central nervous system (CNS) infarction of the brain attributable to ischemia in a defined vascular distribution. ICH was diagnosed if there was a focal collection of blood within the brain parenchyma or ventricular system that was not caused by trauma. SAH was diagnosed if there was bleeding into the subarachnoid space.

### 2.6. Investigations

Patients underwent the following investigations: full blood count, renal panel, glucose and electrocardiogram (ECG). Those with ICH or SAH also had prothrombin time (PT) and activated partial thromboplastin time (aPTT) measured. Those with IS had fasting lipids, echocardiography and cardiac monitoring for up to 72 h. 

### 2.7. Ischaemic Stroke Syndrome Classification and Mechanism

For IS, the clinical syndrome was classified as per the Oxfordshire Community Stroke Project [15]—Lacunar Infarct (LACI—pure motor stroke, pure sensory stroke, sensori-motor stroke or ataxic hemiparesis); Total Anterior Circulation Infarct (TACI—higher cerebral dysfunction, e.g., dysphasia, dyscalculia, visuospatial disorder; homonymous visual field defect; ipsilateral motor and/or sensory deficit of at least two areas of the face, arm and leg); Partial Anterior Circulation Infarct (PACI—only two of the three components of TACI syndrome with higher cerebral dysfunction alone or with a motor/sensory deficit more restricted than LACI); Posterior Circulation Infarct (POCI—ipsilateral cranial nerve palsy with contralateral motor and/or sensory deficit; bilateral motor and/or sensory deficit; disorder of conjugate eye movement; cerebellar dysfunction without ipsilateral long-tract deficit; isolated homonymous visual field defect).

The stroke mechanism of IS was categorized using the Trial of Org 10,172 in Acute Stroke Treatment (TOAST) classification [16]—Large Artery Atherosclerosis (LAA—clinical and brain imaging findings of either significant (>50%) stenosis or occlusion of a major brain artery or branch cortical artery, presumably due to atherosclerosis); Cardioembolism (CE—arterial occlusion presumably due to an embolus arising in the heart); Small Artery Occlusion (SAO—one of the traditional clinical lacunar syndromes); Stroke of Other Determined Etiology (SODE—rare causes of stroke, such as non-atherosclerotic vasculopathies, hypercoagulable states or haematologic disorders); Stroke of Undetermined Etiology (SUE—cause of a stroke cannot be determined despite an extensive evaluation, two or more potential causes of stroke or evaluation was cursory).

Patients were managed by the treating physician in consultation with the patient and family. All stroke patients were admitted under the care of the three neurologists (for IS) or the neurosurgeon (for ICH, SAH) working in Raffles Hospital.

### 2.8. Study Processes

The study was performed by the author. Potential subjects were identified by the Information Technology department if they had a diagnosis of stroke, using International Classification of Diseases, Tenth Revision, Clinical Modification (ICD-10-CM) codes I60-I69, and were admitted to Raffles Hospital between 1 June 2015 and 31 December 2023. The case records were reviewed. If the inclusion criteria were met, to respect patient privacy, non-identifiable study data were recorded using standardised forms. Data were entered into a database and subsequently analysed by the author.

### 2.9. Statistical Analysis

Normally distributed continuous data were summarily described by means and standard deviations and non-normally distributed data by medians, while proportions were used for categorical data. To test for associations, an unpaired t-test was used for normally distributed continuous variables and X^2^ for categorical variables. Variables with a significant association on univariable analysis at *p* < 0.1 were then entered into a multivariable logistic regression (model 1). The multivariable logistic regression was then repeated using all variables even if they were not statistically significant on univariable analysis in case there was an effect of showing an apparent borderline statistical significance by reducing the number of factors evaluated (model 2). Associations with *p* < 0.05 were considered statistically significant. Data were analysed using Statistical Package for Social Studies (SPSS) v21.

### 2.10. Ethics

The study was performed in accordance with the Declaration of Helsinki. The study was approved by the Ethics Committee of Raffles Hospital.

## 3. Results

Data were collected on 1165 patients, with a mean age of 65.6 (± 12.9) yr; 47.4% were female and patients were mostly Chinese (83%) with Malay and Indian minorities. HT (63.5%) and HL (60.3%) were the most common vascular risk factors, with SM and DM being among the less common; 24.2% of patients had pCeVD (Table 1). Two females had taken OCP or HRT.

**Figure 1:**
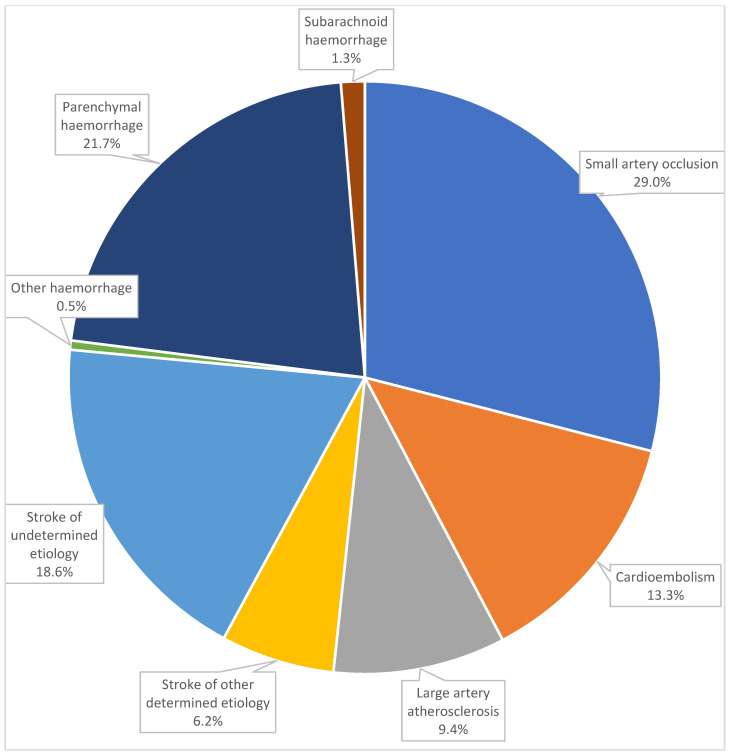
Stroke subtypes and mechanisms (*n* = 1165).

HS (*n* = 274) comprised 23.5% of patients (95%CI 21.1–26.1%) (ICH 21.7%, SAH 1.3%, other haemorrhage 0.5%), while IS (*n* = 891) comprised 76.5% (95%CI 73.9–78.9%) (SAO 29.0%, CE 13.3%, LAA 9.4%, SODE 6.2%, SUE 18.6%) (Figure 1). When only IS was analysed, SAO comprised 37.9% of patients, CE 17.4%, LAA 12.3%, SODE 8.1% and SUE 24.3%; the clinical syndrome was 55.0% LACI, 26.7% TACI, 9.0% PACI and 9.2% POCI.

On univariable analysis, there were significant differences between HS and IS in age (*p* = 0.002), ethnicity (*p* = 0.014), DM (*p* < 0.001), HL (*p* < 0.001), SM (*p* < 0.001) and HD (*p* = 0.009) but not with sex or HT, and there were borderline differences with pCeVD (*p* = 0.096). On multivariable analysis using logistic regression and entering only variables with possibly significant association (*p* < 0.1) on univariable analysis, the association remained for ethnicity (*p* = 0.044), while HS was negatively associated with DM (OR 0.27, 95%CI 0.18–0.41, *p* < 0.001) and SM (OR 0.47, 95%CI 0.34–0.64, *p* < 0.001) and borderline with HD (OR 0.69, 95%CI 0.45–1.06, *p* = 0.088) (model 1) (Table 2). When all variables were entered into the logistic regression model even if they were not significantly associated on univariable analysis, there was an increase in the significant association with ethnicity (*p* = 0.029), new significant positive association with female sex (OR 1.46, 95%CI 1.05–2.02, *p* = 0.024) and HT (OR 1.70, 95%CI 1.13–2.56, *p* = 0.011), additional significant negative association with HL (OR 0.59, 95%CI 0.38–0.94, *p* = 0.026), continued borderline association with HD (OR 0.68, 95%CI 0.44–1.04, *p* = 0.077) and a lack of association with pCeVD (model 2) (Table 2).

Inter-ethnic comparisons of demographics, vascular risk factors and stroke mechanism of all strokes combined revealed significant differences in age (*p* < 0.001, lowest in others), female sex (*p* = 0.005, lower in Indians and others) and HS (*p* = 0.014, lower in Indians and others), borderline differences with DM (*p* = 0.07) and HD (*p* = 0.07), and no significant differences in HT, HL, SM and pCeVD (Table 3).

When IS (*n* = 891) was compared between ethnicities, there were significant differences in age (*p* = 0.001, lower among others) and sex (*p* = 0.016, fewer females in Indians and others) (Table 4). There were no significant differences in vascular risk factors such as HT, DM, HL, SM, HD or pCeVD. When IS was compared, there were no significant inter-ethnic differences whether the TOAST criteria (*p* = 0.103) or OCSP classification (*p* = 0.31) was used.

**Table 4:** Inter-ethnic comparison of patient demographics, vascular risk factors, stroke syndromes and mechanisms of ischaemic stroke.

	This Study				*p*	Sharma, et al. [17]			*p*	De Silva, et al. [18]		*p*
Year	June 2015 to December 2023					September 2003 to August 2004				2001–2003		
N	891					481				2751 of 3096 (including 367 TIAs)		
Age (yr)												
Mean (SD)	65.9 (12.5)					64.1 (11.9)						
Median	66.3											
Female (%)	47.5					40.3				44.4		
Ethnicity	Chinese	Malay	Indian	Others		Chinese	Malay	Indian		Chinese	South Asian	
N (%)	730 (81.9)	76 (8.5)	78 (8.8)	7 (0.8)		357 (74.2)	80 (16.6)	44 (9.2)		2554 (incl 332 TIA) (82.5)	197 (incl 35 TIA) (6.4)	
Age (yr)												
Mean (SD)	66.7 (12.3)	61.1 (11.8)	63.7 (12.9)	57.7 (20.9)	**0.001**	64.0 (11.9)	66.6 (10.8)	60.2 (12.8)	0.015			
Median	67.0	62.7	65.7	58.2						67	62	**<0.001**
Female (%)	49.3	47.4	33.3	14.3	**0.016**	42.9	31.2	36.4	0.14	46.0	23.8	**<0.001**
Hypertension (%)	64.4	61.8	56.4	57.1	0.543	83.2	85.0	84.1	0.92	77.0	75.1	0.479
Diabetes mellitus (%)	36.3	36.8	48.7	28.6	0.18	39.8	67.5	52.3	**<0.001**	41.0	59.9	**<0.001**
Hyperlipidaemia (%)	63.6	64.5	71.8	57.1	0.524	76.5	78.8	86.4	0.321	40.0	51.8	**<0.001**
Smoking (%)	38.5	44.7	34.6	42.9	0.619	11.8	20.0	15.9	0.133	25.0	35.0	**0.004**
Ischaemic heart disease (%)	17.1	23.7	25.6	14.3	0.171	32.8	37.5	38.6	0.579	22.0	40.1	**<0.001**
Previous CeVD	26.7	21.8	17.9	14.3	0.244	-	-	-	-	-	-	-
Subtype (Excluding TIA) (OCSP)					0.31	-	-	-				‘similar’
TACI	25.5	39.5	25.6	28.6						6.9	6.2	
PACI	8.5	5.9	14.1	14.3						13.8	14.8	
POCI	9.5	7.9	7.7	14.3						11.5	12.3	
LACI	56.6	44.7	52.6	42.9						67.8	66.7	
Subtype (TOAST)					0.103				**0.017**	-	-	-
LAA	12.3	6.6	16.7	14.3		12.3	17.5	25.0				
CE	17.9	17.1	14.1	0		8.4	7.5	4.5				
SAO	38.8	32.9	34.6	42.9		51.8	42.5	25.0				
SODE	7.7	10.5	9.0	14.3		2.0	2.5	6.8				
SUE	22.9	32.8	25.6	28.6		25.5	30.0	38.6				

Legend: N = number, SD = standard deviation, TOAST = Trial of Org10172 in Acute Stroke Treatment, LAA = Large Artery Atherosclerosis, CE = Cardioembolism, SAO = Small Artery Occlusion, SODE = Stroke of Other Determined Aetiology, SUE = Stroke of Undetermined Aetiology, OCSP = Oxfordshire Community Stroke Project, TACI = Total Anterior Circulation Infarct, PACI = Partial Anterior Circulation Infarct, POCI = Posterior Circulation Infarct, LACI = Lacunar Infarct. Statistics: to test for associations, an unpaired *t*-test was used for normally distributed continuous variables and X^2^ for categorical variables.

## 4. Discussion

This study showed that acute stroke patients in multi-ethnic Singapore are elderly and slightly more likely to be male, with hypertension, hyperlipidaemia and possibly diabetes mellitus, and a smoker. The main stroke mechanism is infarction, and the ischaemic stroke subtype is due to small artery occlusion and lacunar syndrome. Compared to IS, HS is negatively associated with diabetes mellitus and smoking. When the ethnicities are compared, there are notable differences in sex and stroke mechanism, with fewer females and haemorrhagic stroke among Indians and others compared to Chinese people and Malays. 

The demographics and risk factor profile found in this study are consistent with the factors in stroke risk factor calculators such as the Framingham Stroke Profile [19]. The data also resemble profiles of stroke risk factors reported from other stroke registries in Asia [6]. The frequency of HT in this study was 63.4% (in other Asian registries it is 50.0–96.4%); the frequency of DM was 31.8% (9.3–50.0%), SM 35.6% (17.0–59.4%), HD 16.8% (11.8–20.2%) and pCeVD 24.2% (13.7–32.3%). However, HL at 60.3% was higher (5.3–45.4%). The patient demographics and risk factor profile in this study are also consistent with the national registries of Singapore [9] (data for 2021, *n* = 9680) (Raffles Hospital did not participate) and Malaysia [10] (for 2010–2014, *n* = 7820) (Table 1). The age of study patients and proportion of females was in between that of Singapore and Malaysia. The frequency of risk factors was lower for HT and DM, in between for HL and SM but higher for HD and pCeVD. As expected, the ethnic profile of this study resembled that of the Singaporean registry—both registries reflect the ethnic profile, demographics and risk factor profiles of stroke patients of their respective countries.

The proportion of stroke due to HS in this study (ICH 21.7% + SAH 1.3% SAH + other haemorrhage 0.5% = 23.5%) was less than in the GBD study [1] (ICH 28.9% + SAH 5.9% = 34.8%). The Asian stroke registries reported ICH ranging from 16.1% to 49.1% and SAH from 1.3 to 8.0% [6]. IS in this study was 76.5%, and it was 65.5% in the GBD study [1] and 42.9%–80.0% in the Asian registries [6]. The frequency of HS was 18.2% in both the Singaporean and Malaysian registries [9,10] (Table 1). The higher frequency of HS in this study may be a reflection of the stroke subtypes of patients admitted to Raffles Hospital. 

There may be different risk factors for HS compared to IS. INTERSTROKE was a case–control study involving 22 countries that included 663 HS and 2337 IS patients [20]. It reported that the prevalence of HT was 60% in HS and 55% in IS; DM 10% vs. 21%; SM 31% vs. 37%. The Singapore [9] and Malaysia [10] registries showed similar frequencies of HT between HS and IS but a lower frequency of DM, HL and SM, and for Malaysia, a lower frequency of HD in HS compared to IS (Table 1). In this study, there were significantly lower odds of DM (OR 0.27) and SM (OR 0.47) in HS vs. IS; when all variables were included, there were increased odds of female sex (OR 1.46) and HT (OR 1.70) and an additional significant negative association with HL (OR 0.59). These findings are in line with what was reported in INTERSTROKE [20] and the two national registries [9,10]. This is consistent with the known pathophysiology of HS (the rupture of arteries due to HT (usually microaneurysms)) and IS (usually atherosclerosis of small or large arteries due to HT, DM, HL or SM [21]).

However, risk factor differences alone may not explain the differential risk of developing in HS or IS in a person. As mentioned before, the highest incidence of ICH was in Oceania, East Asia, Central Asia and Southeast Asia, while the lowest was in Western Europe and Australasia [2,5]. This possibility of inter-ethnic differences in stroke subtypes was explored in a systematic review comparing stroke and its subtypes in Chinese compared to white populations [22]. It showed that ICH accounted for a larger proportion of strokes in Chinese than white studies (pooled proportion 33% vs. 12%). The proportion of lacunar ischemic stroke was higher in Chinese than white populations. A subsequent systematic review and meta-analysis was performed of risk factors for IS and its subtypes in Chinese compared to Caucasians (7 studies involving 16,199 Chinese and 11 involving 16,189 Caucasians) [23]. Chinese patients had a younger onset of stroke, similar prevalence of hypertension, diabetes mellitus, smoking and alcohol, and significantly lower prevalence of atrial fibrillation, ischemic heart disease and hypercholesterolemia. Risk factor associations with IS subtypes were mostly similar among Chinese and Caucasian patients. However, the authors felt that studies using comparable, case-ascertainment and classification methods, as well as further analyses of individual patient data with adjustment for confounders, were needed.

A more recent and broader systematic review and meta-analysis of the association of modifiable risk factors with IS subtypes in Asian versus Caucasian populations included 32 studies with a total of 23,404 IS patients (14,364 Asians, 9040 Caucasians) [24]. Subgroup analysis based on ethnicity revealed a significant association with dyslipidemia, diabetes mellitus and atrial fibrillation in large artery atherosclerosis (LAA) for both Asians and Caucasians. Hypertension was significantly associated with Small Vessel Occlusion (SVO) and Stroke of Other Determined Aetiology (SODA) in both Asians and Caucasians; however, only Asians showed a significant association of hypertension in LAA and cardioembolism (CE). This study suggests that there may be differing associations between stroke risk factors and IS subtypes depending on ethnicity.

Further insights into inter-ethnic differences may be obtained from intra-country studies that use standardised case ascertainment, data collections forms, definitions and classifications while they specifically investigate inter-ethnic differences. A recent Irish stroke unit study comparing 44 patients originally not of Irish ethnicity with 437 ethnic Irish found a younger age (mean age 57.5 ±13 yr vs. 69.6 ± 13.2 yr, *p* < 0.001), higher proportion of males (75% vs. 57.4%, *p* = 0.02) and ICH (34.1% vs. 11.7% *p* < 0.01) [25]. A community-based stroke study in New Zealand (*n* = 1,119,192) revealed ethnic differences in the distribution of stroke subtypes, with the highest proportion of IS observed in Europeans (83%), the highest proportion of ICH in Asians (22%) and the highest proportion of SAH in Māori (14%) [26]. A nationwide register-based cohort study (*n* = 7,423,174) in the Netherlands showed that Surinamese men and women had a higher (adjusted hazard ratio aHR 1.43, 95%CI 1.35–1.50; aHR 1.34, 95%CI 1.28–1.41, respectively) risk of stroke of all types compared to ethnic Dutch incidence rates of all stroke subtypes combined, while Moroccan men and women had a lower (aHR 0.42, 95%CI 0.36–0.48; aHR 0.37, 95%CI 0.30–0.46) risk; these risk differences persisted for the various subtypes of stroke—IS, ICH and SAH [27]. In a hospital-based registry study in South Africa (*n* = 524), black patients were younger (mean age 51) than white patients (mean age 51 yr vs. 61 yr) and had more cerebral hemorrhage (27% vs. 15%), lacunar infarcts (28% vs. 22%) and total anterior circulation infarcts (28% vs. 22%); large vessel atherosclerosis was not detected [28]. In a study in the United Kingdom (*n* = 1200), compared to whites, black patients with stroke were younger, more likely to have a stroke due to cerebral small vessel disease (33% vs. 14%) and less likely to have large vessel atherosclerosis (OR 0.49; 95%CI 0.29–0.82; *p* = 0.007) and cardioembolic disease (OR 0.54; 95%CI 0.37–0.80; *p* = 0.002) [29]. In the United States of America in a pooled cohort of the Atherosclerosis Risk in Communities Study (ARIC) and the Cardiovascular Health Study (CHS) (*n* = 15,792 and 5888, respectively) with over 263,489 person-years of follow-up, African American ethnicity was a risk factor for incidental ICH [30]. In a study of Asian Americans with whites as a reference, the adjusted relative risk (95%CI) of all Asians for HS was 1.6 (95%CI 1.1–2.3, *p* = 0.01) due to increased risks of SAH in Japanese people and ICH in Filipinos [31]. Some studies attributed these differences to differences in stroke risk factors [2,28] though genetic susceptibility has also been suggested [29].

The previously mentioned systematic review comparing stroke and its subtypes in Chinese people compared to white populations showed that ICH accounted for a larger proportion of strokes in Chinese than white studies [22]—the Chinese had a lower prevalence of HL and HD, but that may not fully explain the difference in HS vs. IS between the two ethnicities as genetic susceptibility may have a role [29]. This study has further explored this. There was a suggestion of inter-ethnic differences in stroke mechanism (HS vs. IS) in the raw data (Table 1 and Table 3), which was further explored by logistic regression (Table 2)—even after adjusting for risk factors, there still was a significant difference—that was attributable to ethnicity and became even more significant (*p* = 0.029) when all the vascular risk factors were entered into the model and adjusted for.

When only IS was analysed, the median age in this study was slightly younger at 66.3 years compared to 70.3 years in Singapore [9]; the mean age was 65.9 years, which was similar to 63.2 years in Malaysia [10] (Table 1). Females in this study were slightly higher at 47.3% compared to 44.8% in Malaysia. As expected, the predominant ethnicity in this study was Chinese (81.9%), while it was Malay (88.7%) in Malaysia [10], reflecting the ethnicities of the respective countries. The differences in risk factors are shown in Table 1. Overall, the frequency in this study was lowest for HT and DM and in between Singapore [9] and Malaysia [22] for HL and SM. The clinical syndrome among IS in this study was 55.0% LACI, 26.7% TACI, 9.0% PACI and 9.2% POCI; in INTERSTROKE [20], it was 21% LACI, 8% TACI, 52% PACI and 14% POCI, with similar findings among patients from Southeast Asia (*n* = 1146; China, Malaysia, Philippines). The reason for these differences is unclear. SAO was the most frequent mechanism of IS by TOAST criteria in this study at 37.9%, while CE was 17.4%, LAA 12.3%, SODE 8.1% and SUE 24.3%. A smaller multi-ethnic hospital-based study in Singapore (*n* = 481, fewer females at 40.3% vs. 47.5% in this study and a similar mean age of 64.1 ± 11.9 years vs. 65.9 ± 12.5 years) showed that the most frequent mechanism for IS was SAO at 47.9%, while CE was 7.9%, LAA 14.3%, SODE 2.5% and SUE 27.4% [17]. In INTERSTROKE [20], the frequency of SAO was 44%, while CE was 9%, LAA 19%, SODE 5% and SUE 22%. Again, the reason for these differences is unclear.

Inter-ethnic comparisons in IS showed significant differences in age (*p* = 0.001) and sex (*p* = 0.016) but no significant differences in vascular risk factors, stroke syndrome (*p* = 0.31) or stroke mechanisms (*p* = 0.103). The earlier mentioned smaller multi-ethnic hospital-based study in Singapore also found differences in age (*p* = 0.015) and DM (*p* < 0.001) but not in sex or other risk factors (Table 4); however, interethnic differences in stroke subtype were found (*p* = 0.017) [17]. Another Singapore hospital-based study (*n* = 3096) that included IS and TIAs compared Chinese with South Asians and found significant differences in age, sex, DM and HD—any differences in HL and SM disappeared on regression analysis; stroke syndromes were similar between the two ethnicities, similar to this study [18]. The apparent differences between the three studies warrant further study.

Disparities have been attributed to differing statuses of countries, which are strongly associated with the occurrence and management of modifiable risk factors [3]. Only middle-income countries experienced rising trends in age-standardised stroke prevalence despite falling trends in age-standardised stroke incidence and mortality since 2005. The reduction in stroke death rate by almost 34% attributable to modifiable risk factors was more prominent in wealthier countries. 

The availability of and access to healthcare services, attitudes and lifestyles may add to the complex interplay between RF and ethnicity. In South Western Sydney, which has a culturally and linguistically diverse and lower socioeconomic status population, pre-hospital delay was seen more among those who were younger, who did not use ambulance services or who belonged to Polynesian, South Asian and Mainland Southeast Asian ethnicities [32]. In Denmark, immigrants had a longer prehospital delay than Danish-born residents [33]. In New York, Hispanic and African American women had delayed hospital arrival times (≥3 h) after stroke symptom onset, but this difference between races or ethnicities was no longer present after adjusting for socioeconomic status [34]. In South London, patients of black ethnicity had increased odds of delay [35]. These arrival delays may be mitigated by in-hospital system interventions to reduce door-to-needle (DTN) time, symptomatic intracerebral haemorrhage and mortality [36].

Considering genetics in the development of disease [37,38], single-gene disorders may be the cause of rare stroke disorders eg Cerebral Autosomal Dominant Arteriopathy with Subacute Ischaemic Leucoencephalopathy (CADASIL) and moyamoya disease [39]. But common and rare genetic polymorphisms can influence the risk of more common causes of stroke, both due to other RF or stroke-specific mechanisms. Only half of the excess stroke risk among blacks in the United States is attributable to traditional risk factors—the pro-inflammatory marker interleukin-6 (IL-6) and the highly genetically-determined lipoprotein [a] (Lp(a)) are higher in some ethnicities and may elevate stroke risk [40]. An example of the inter-play of ethnicity with risk factors was seen in a study where a diet high in saturated fat was associated with a lowered risk of ICH by 45% and to a lesser extent of IS by 18% among Japanese but not non-Japanese [41]. But we are cautioned by a meta-analysis (*n* = 32,431–12,883 cases and 19,548 controls) involving people of Chinese, Japanese and Korean descent with gene variants of angiotensin-I converting enzyme (ACE) insertion/deletion (I/D) polymorphisms, the C677T variant of 5,10-methylenetetrahydrofolate reductase (MTHFR) and the apolipoprotein E (APOE) gene not showing an increased association with IS compared with persons of European descent [42]. The prospective multi-ethnic SingHEART study of Chinese, Malays and Indians by questionnaire, cardiac evaluation, lipidomics and whole genome sequencing to assess stroke, myocardial infarction and other outcomes should provide valuable insight into RF, ethnicity and genetics on vascular outcomes [43].

There are a few limitations in this study. This is a single-centre study with a modest sample size. Non-Chinese ethnicities were relatively under-represented. Data were not collected on obesity, exercise, alcohol intake or dietary habits. There were no data on adequacy of risk factor control or compliance with medications prior to a stroke. The study is limited to a specific geographical location (Singapore)—this may not represent the global population’s stroke patterns, limiting the generalisability of the findings. Moreover, the study did not consider the impact of genetic factors on stroke risk among different ethnicities. Genetic predispositions can significantly influence stroke risk and may vary among different ethnic groups. Cultural factors, such as stress levels and lifestyle habits, can also influence stroke risk and may vary among different ethnic groups. Moreover, the study does not account for potential changes in stroke risk factors and treatments over time. However, this study has numerous strengths. It involved a well-defined study population that resembled the profile of stroke patients in the national stroke registry of Singapore, allowing extrapolation of study findings to the national population. The three main ethnicities were included. Data were collected in a standardised manner. This study is more recent than the previous two Singapore IS studies carried out two decades ago that did not include HS and has double the sample size of the more recent of the studies. IS was analysed by both OSCP classification and TOAST criteria. Most importantly, it provided much-needed and novel data on the significance of inter-ethnic differences in stroke mechanisms among Asians, adding new knowledge to this controversial field—this will allow a better understanding of ethnicity and stroke. While differences in healthcare systems and access can significantly impact stroke risk and outcomes, this is unlikely to have a significant impact here as highly subsidised healthcare is widely available and accessible in Singapore.

There are important policy and practice implications of the study findings. Stroke patients are elderly, consistent with most studies, and predominantly Chinese, which is to be expected as they comprise the largest ethnic group in Singapore. HT and HL are the most common modifiable stroke RF—programs to reduce stroke risk in Singapore should target these two diseases; however, SM should not be neglected. IS is the predominant stroke mechanism and is generally less severe than HS. IS is most commonly due to SAO and lacunar syndrome predominates—these are generally milder strokes and carry a better prognosis for recovery. Hypertension was similarly associated with IS and HS in the initial analysis but strongly associated with HS when all variables were entered into the analysis—a history of hypertension may, thus, indicate HS rather than IS. But the presence of DM, HLD or SM suggests IS rather than HS. 

The significant association of stroke subtype with ethnicity is the novel finding of this study. Based on this, further research on inter-ethnic differences can be performed. The role of factors not evaluated in this study needs to be investigated, e.g., obesity, exercise, diet, obstructive sleep apnoea, genetics, socioeconomic status, chronic infections and inflammation markers. Larger numbers of subjects are needed. Different populations with similar ethnicities should be involved. This can be achieved by multi-centre, multi-national studies with a large number of subjects involving many countries with similar ethnicities (e.g., Indonesia, Malaysia, Singapore, in this example) and using standard definitions and case report forms with centralised analysis.

## 5. Conclusions

Stroke is a major cause of disease globally. Socioeconomic factors and risk factor differences may not fully explain disparities in stroke burden. The possibility of contributions due to ethnicity have been hinted at by inter-country ecological studies and substantiated by intra-country comparisons. This study comprising Chinese people, Malays, Indians and others living in Singapore showed that the patients were elderly, with hypertension and hyperlipidaemia, and with the majority having ischaemic stroke with lacunar as the most common clinical syndrome and small artery occlusion as the most frequent mechanism among ischaemic stroke patients. When haemorrhagic and ischaemic stroke were compared, even after adjusting for differences in stroke risk factors, the role of ethnicity remained significant. No inter-ethnic differences were found in ischaemic stroke syndrome or mechanism, possibly due to the study sample size. Multi-centre, multi-ethnic studies with large sample sizes would be needed to corroborate the findings of this study and to uncover additional reasons for these differences. The findings may help guide ethnicity-specific stroke prevention and management policies and practice.

## Figures and Tables

**Table 1:** Patient demographics, vascular risk factors and stroke subtype.

	This Study			Singapore Stroke Registry			Malaysian Stroke Registry (1)		
Year	2015–2023			2021			2010–2014		
	Overall	Haemorrhage	Infarct	Overall	Haemorrhage	Infarct	Overall	Haemorrhage	Infarct
N, %	1165	23.5	76.5	9680	18.2	81.8	7830 (2)	18.2	79.4
Age (yr)									
Median	66.3	66.4	66.3	69.8	66.8	70.3			
Mean (SD)	65.6 (12.9)	64.7 (14.1)	65.9 (12.5)				62.7 (12.5)	61.2 (12.9)	63.2 (12.4)
Female (%)	47.4	47.1	47.5	41.9	ND	ND	44.9	45.5	44.8
Ethnicity (%)					ND	ND			
Chinese	83.0	86.5	81.9	74.6			(3)	(3)	(3)
Malay	8.8	9.9	8.5	14.8			8.3	7.2	8.6
Indian	7.4	2.9	8.8	7.5			89.2	91.5	88.7
Others	0.8	0.7	0.8				2.5	1.3	2.7
Hypertension (%)	63.4	63.5	63.4	80.6	80.6	80.6	70.0	73.5	69.2
Diabetes mellitus (%)	31.8	13.5	37.4	43.1	30.1	46.0	41.5	26.2	45.0
Hyperlipidaemia (%)	60.3	47.1	64.3	83.2	60.7	88.1	29.5	23.4	30.9
Smoking	35.6	25.5	38.7	34.0	26.8	35.5	54.8	52.2	55.4
Ischaemic heart disease (%)	16.8	11.7	18.4	ND	ND	ND	11.9	6.2	13.2
Previous cerebrovascular events (%)	24.2	20.4	25.4	ND	ND	ND	20.8	ND	ND

Legend—N = number, SD = standard deviation. (1) Includes only classified stroke, unless stated otherwise. (2) Includes 2% TIA, 0.4% unclassified stroke type. (3) Includes only Chinese, Malays and Indians.

**Table 2:** Patient demographics and vascular risk factors of haemorrhagic versus ischaemic stroke.

					Model 1		Model 2	
	Overall	Haemorrhage	Infarct	*p*	Adjusted OR (95%CI)	*p*	Adjusted OR (95%CI)	*p*
N, %	1165	23.5	76.5					
Age, mean (SD) (yr)	65.6 (12.9)	64.7 (14.1)	65.9 (12.5)	0.002	0.99 (0.98–1.01)	0.30	1.00 (0.99–1.01)	0.88
Female (%)	47.4	47.1	47.5	0.91	-		1.46 (1.05–2.02)	0.024
Ethnicity (%)				0.014		**0.044**		**0.029**
Chinese	83.0	86.5	81.9					
Malay	8.8	9.9	8.5					
Indian	7.4	2.9	8.8					
Others	0.8	0.7	0.8					
Hypertension (%)	63.4	63.5	63.4	0.98	-	-	**1.70 (1.13–2.56)**	**0.011**
Diabetes mellitus (%)	31.8	13.5	37.4	<0.001	**0.27 (0.18–0.41)**	**<0.001**	**0.31 (0.20–0.48)**	**<0.001**
Hyperlipidaemia (%)	60.3	47.1	64.3	<0.001	0.90 (0.64–1.26)	0.53	**0.59 (0.38–0.94)**	**0.026**
Smoking	35.6	25.5	38.7	<0.001	**0.47 (0.34–0.64)**	**<0.001**	**0.40 (0.28–0.56)**	**<0.001**
Ischaemic heart disease (%)	16.8	11.7	18.4	0.009	0.69 (0.45–1.06)	0.088	0.68 (0.44–1.04)	0.077
Previous cerebrovascular events (%)	24.2	20.4	25.4	0.096	0.87 (0.61–1.24)	0.44	0.81 (0.57–1.16)	0.25

Model 1—multivariable logistic regression performed using variables significant at *p* < 0.1 in the univariable analysis. Model 2—multivariable logistic regression performed using all variables in the univariable analysis. Legend—N = number, SD = standard deviation.

**Table 3:** Inter-ethnic comparison of patient demographics, vascular risk factors and stroke subtype.

Ethnicity	Chinese	Malay	Indian	Others	*p*
N (%)	967 (83.0)	103 (8.8)	86 (7.4)	9 (0.8)	
Age (yr)					
Mean (SD)	66.2 (12.7)	61.8 (13.1)	64.0 (12.8)	54.7 (20.7)	**<0.001**
Median	66.9	63.6	66.0	58.2	
Female (%)	49.3	44.7	31.4	22.2	**0.005**
Hypertension (%)	64.0	64.1	58.1	44.4	0.46
Diabetes mellitus (%)	30.3	32.0	44.2	22.2	0.07
Hyperlipidaemia (%)	59.5	62.1	68.6	44.4	0.28
Smoking (%)	35.4	40.8	32.6	33.3	0.66
Ischaemic heart disease (%)	15.6	22.3	24.4	11.1	0.07
Previous CeVD (%)	25.0	22.3	18.6	12.2	0.41
Haemorrhagic stroke (%)	24.5	26.2	9.3	0.7	**0.014**

Legend: N = number, SD = standard deviation, CeVD = cerebrovascular disease. Statistics: to test for associations, an unpaired *t*-test was used for normally distributed continuous variables and X^2^ for categorical variables.

## Data Availability

Data are available from the author on reasonable request.
